# Mobile time banking on blockchain system development for community elderly care

**DOI:** 10.1007/s12652-022-03780-6

**Published:** 2022-03-15

**Authors:** Hungyi Chen, Yuan-Chia Chu, Feipei Lai

**Affiliations:** 1 Graduate Institute of Biomedical Electronics and Bioinformatics, National Taiwan University, Taipei, 10617 Taiwan; 2 Information Management Office, Taipei Veterans General Hospital, Taipei, 11217 Taiwan; 3 Department of Computer Science and Information Engineering, National Taiwan University, Taipei, 10617 Taiwan; 4 Department of Electrical Engineering, National Taiwan University, Taipei, 10617 Taiwan

**Keywords:** Blockchain, Time banking, Elderly care, Mobile health, Employee volunteerism, Sustainable development goals

## Abstract

This study aims to develop a mobile time-banking system on blockchain (MTBB), which can track service transaction records for community elderly care via mutual service exchange. The MTBB was developed to enable organizations, either corporate-social-responsibility organizations or nonprofit organizations to issue proprietary time tokens to members who participate in the organizations’ volunteer activities. Database applications with smartphone apps integrated with MultiChain blockchain technology were developed. Metadata with the service transaction information are stored in the MultiChain blocks so that the transaction records are immutable and can be analyzed in the future. Cahn’s time-banking guidelines were applied in developing this MTBB with MultiChain blockchain technology integrated for tracking service transaction records. The study also combines one-to-one mutual service exchange with organizations which offer volunteer activities and issue proprietary time tokens. With the blockchain transaction tracking mechanism, all elderly care service records via or within organizations can be tracked and analyzed to show their alignment with some of the Sustainable Development Goals of the United Nations.

## Introduction

In addition to social welfare and out-of-pocket fee payment mechanisms, time banking is an effective mechanism for providing elderly care as a community service, in which members provide mutual benefits. With further integration that enables it to capture offline work, mobile time banking can provide a better system than traditional web access. However, there have been difficulties in integrating blockchain technology with real-world economies or activities. The key idea of this study is to provide a blockchain-based mobile business application system for organizations to better manage community elderly care services, with the service records being immutable.

The objective of this paper is to develop a mobile time banking system on blockchain (MTBB) by following Cahn’s time-banking mechanism guidelines and leveraging the immutability feature of blockchain technology on smartphones for elderly care service exchanges. When tracking elderly care services on blockchain, data immutability will create a trusted foundation for further data analysis in communities. The use of MultiChain blockchain technology in the MTBB requires neither block pre-allocation nor mining. Thus, it does not waste energy because issuing coins by mining is notorious for its consumption of resources or energy. Organizations, either volunteer-involving organizations (VIOs) or corporations with employee volunteerism (EV), can use the MTBB to track volunteer activities and issue their proprietary time tokens to their participating volunteer members. Members can then use the proprietary time tokens that they have earned to access elderly care services within organizations, while the organizations have the role of coordinating the activities in the local time banks. All elderly care service transaction data are put on blockchain and can be tracked so that the organizations can measure their Sustainable Development Goal (SDG) achievements. The development of the MTBB may provide a realistic solution for community elderly care. In addition, the tracking mechanism from blockchain technology can help track elderly care service transaction records to better measure how well the SDGs set by the United Nations (UN) are satisfied.

The contributions of this study are as follows.

1. A mobile time-banking system is designed and implemented by integrating blockchain technology so that the community elderly care service records are immutable.
2. This MTBB is designed to provide organizations with the ability to manage both one-to-one mutual service exchange processes and volunteer activity processes in elderly care.
3. The volunteer work recordings of elderly care in this MTBB can help organizations report their SDG achievements.
4. This MTBB is operated in the Software-as-a-Service (SaaS) model so that VIOs or corporations with EV can deploy this management system and issue their proprietary time tokens easily.
5. This MTBB is rolled out to selected organizations in Taiwan so that they can track their elderly care service records easily.

The rest of the paper is organized as follows. Section 2 reviews the related work. Section 3 describes the time-banking guidelines, time token use in business scenarios, and the technology stack to design the system. Section 4 shows the design and implementation of the MTBB by examining business roles, modules, and the business processes involved. Section 5 presents the system rollouts and discussion. Section 6 concludes the paper.

## Related work review

### Time banking

Time banking was introduced by Edgar Cahn in the mid-1980s to enable people to earn time dollars (with an hour equal to a time dollar), bank them, and then use these banked time dollars to “purchase” help from another member. This mechanism requires a computerized system and a coordinator (Cahn 2004). Many explorations of the concept followed, especially regarding equal time, equal value, and the impacts on societies (Avital et al. 2015; Collom et al. 2012; Laamanen et al. 2015; Shih et al. 2015). The time-banking mechanism was studied not only in the US but also worldwide (Boyal and Institute 2014; Glynos and Speedb 2012; Hayashi 2012). Although Cahn developed a traditional web system to promote time banking, there is still limited use of mobile applications for elderly care (Carroll et al. 2016; Han et al. 2014, 2015; Han 2015). Given the current popularity of mobile technology and the mutual community services that occur in the field, usually in contexts that lack immediate access to laptop or desktop computers, application software that works only on the web may suffer from slow adoption. In this study, a time-banking system was developed on smartphones to enable end users to address mobile needs. The coordinating role in time banking has traditionally been held by an individual, but recently, researchers have started exploring how organizations can coordinate (Carroll et al. 2017; Luckner et al. 2015). This paper introduces a design in which organizations hold the coordinator role to better sustain local time-banking operations.

### Blockchain technology

Blockchain is a distributed database that keeps a chronologically growing list (chain) of records (blocks) secure from tampering and revision (i.e., immutable). A record is any piece of evidence about the past. The blockchain (distributed ledger) is a record of all transactions to date (O’Donoghue et al. 2019; Zheng et al. 2017). Blockchain technology has recently been applied to the healthcare domain (Azaria et al. 2016; Hölbl et al. 2018; Ichikawa 2017; Khurshid and Gadnis 2019; Maslove et al. 2018; Meinert et al. 2019; Park 2019), but it is rarely applied to time-banking application software development (Bai and Synnes 2018). In this study, a time dollar is represented by a token on blockchain, i.e., a time token.

### Time banking for elderly care

Governments are facing budget deficits for social welfare, and family members are experiencing rising care costs. Time banking for elderly care has been explored as an alternative. Several aspects of research in this area explore coproduction, social networks, and social capital (Glynos and Speedb 2012; Gregory 2012; Witjaksono 2016). Apparently, time banking for elderly care is the third choice among the typical government social welfare approach, out-of-pocket fee approach and time banking.

### Sustainable development goals of the United Nations

The SDGs of the UN were formally adopted by all member states in 2015 for the 2016–2030 period to address the overwhelming empirical and scientific evidence that the world needs a radical new approach to sustainability. The seventeen SDGs are linked to the five areas of critical importance, including People, Planet, Prosperity, Peace and Partnerships, of which the People area is linked to SDG 1 (No Poverty), SDG 2 (Zero Hunger), SDG3 (Good Health and Well-being), SDG 4 (Quality Education), SDG 5 (Gender Equality), and SDG 6 (Clean Water and Sanitation) (Morton et al. 2017). In this study, the first five SDGs are related to elderly care. Scholars have also explored the interactions among the SDGs (Nilsson et al. 2018; Nunes et al. 2016). In SDG 3, health equality is the key goal of universal health coverage (Rasanathan and Diaz 2016; Tangcharoensathien et al. 2015). Entrepreneurship or social innovation may improve the achievement of these SDGs (Eichler and Schwarz 2019; Schaltegger et al. 2018). Furthermore, with the introduction of information and communication technologies (ICT), SDG achievements can be tracked more effectively (Bebbington and Unerman 2018; Gössling and Hall 2019).

### Comparisons

Existing studies on the mobile use of time banking for elderly care are limited with few real-world rollouts (Cui et al. 2019; Lee et al. 2020). Furthermore, several areas are rarely addressed in current research: the integration of innovative blockchain technology with time banking to achieve data immutability, the integration of volunteer activities with time banking from the business applications perspective for providing multi-process functions, the alignment of time-banking applications for elderly care with SDGs, and the provision of SaaS for easy system deployment by organizations. The comparisons of the MTBB to major related works are listed in Table 1.

**Table 1:** Comparisons of the MTBB to major related works

Related work	Data immut-ability	Multi-process functions	SDG align-ment	Easy deploy-ment	Real-world rollouts
Han et al. (2014, 2015)				X	X
Carroll et al. (2016, 2017)				X	X
Nunes et al. (2016)			X		
Schaltegger et al. (2018)			X		
Cui et al. (2019)	X				
Lee et al. (2020)	X				
Chen et al. (this MTBB)	X	X	X	X	X

## Methods

### Cahn’s time-banking guidelines

The MTBB was developed by following Cahn’s twelve time-banking (TB) guidelines, which are listed in Table 2 (Cahn 2004).

**Table 2:** Cahn’s twelve time-banking guidelines

Cahn’s time-banking guidelines	Description
TB1	Members list the services they can offer and those that they need
TB2	All agree to both give and receive services
TB3	Everyone is interviewed and provides references
TB4	Every hour of giving help earns the giver one credit: a time dollar
TB5	Members ‘buy’ the services they need with their credits
TB6	The computer matches the task, the giver and the receiver
TB7	Every transaction is recorded in a computer ‘time bank’
TB8	Members receive a regular ‘bank’ statement
TB9	One hour is one credit regardless of the skills one offers
TB10	Members can donate credits to friends or to the ‘credit pool’
TB11	Everyone is seen as special, with a contribution to make
TB12	All activities maintain set standards

### MTBB time token use scenarios

**Fig. 1:**
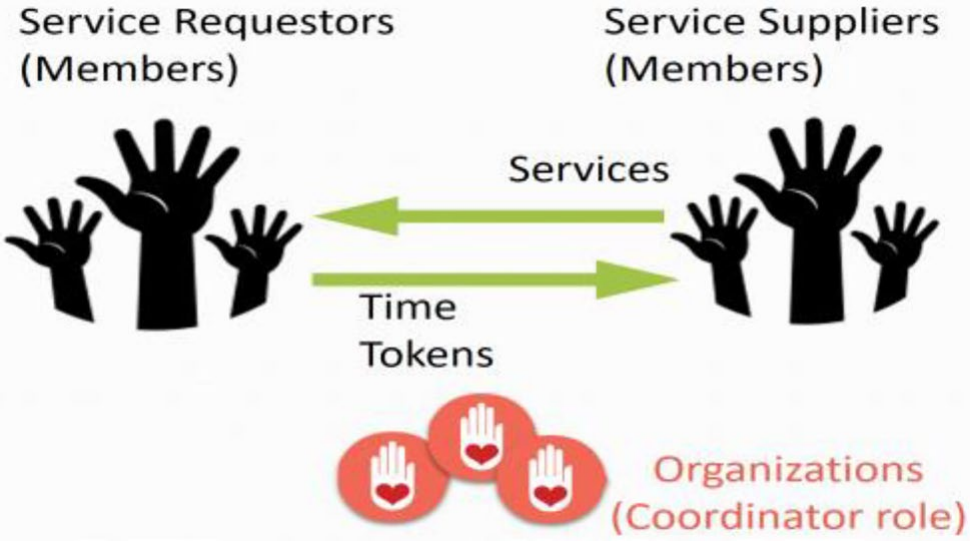
One-to-one member mutual service exchange

When a member has time tokens and would like to exchange them for elderly care services, a time token can be exchanged for one hour of service. The organization has the coordinator role, as illustrated by Cahn, by using the MTBB web backend modules. All service categories are predefined, and an organization can choose the service categories for which it would like to provide services. The organization is responsible for training or verifying that its members have the skills that they need for the service categories. A member can provide services only if he or she has verified service category skills. Members within the same organization, not across organizations, provide services to each other, and a member can participate in multiple organizations, as illustrated in Fig. 1.

**Fig. 2:**
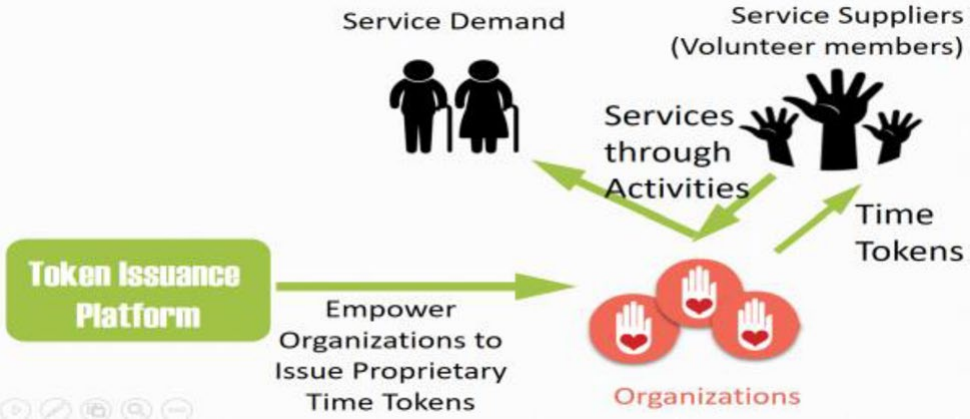
Organizations issuing proprietary time tokens to their volunteer members

Some organizations, either VIOs or corporations with EV, hold frequent volunteer activities. These organizations should be empowered to issue their proprietary time tokens when they hold their volunteer activities, so that after members contribute their time by providing services to elderly people, they will earn time tokens issued by their organizations, as illustrated in Fig. 2.

**Fig. 3:**
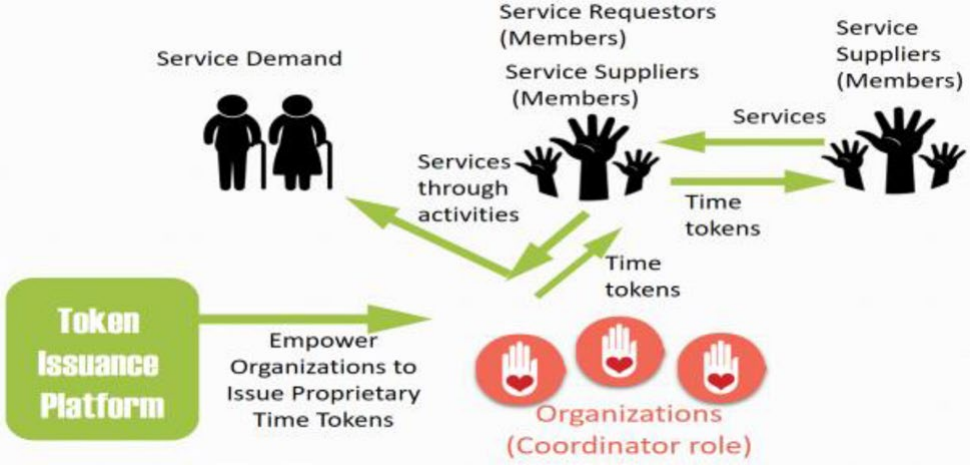
Combined scenario of time token usage

In the combined scenario, members can earn time tokens either by providing elderly care via volunteer activities that are facilitated by their organizations or via one-to-one service provision. Members with proprietary time tokens can then request elderly care services from other members within the same organization. The combined scenario is illustrated in Fig. 3.

### MTBB technology stack

Among the various blockchain technology open stacks, MultiChain was chosen. MultiChain is based on a fork of Bitcoin Core, which supports multicurrency (MultiChain 2019). MultiChain was utilized as a private blockchain in this study. The major technology stack included MultiChain version 1.0.5 with AES-256, MySQL version 5.6.40, Ubuntu version 16.04.1, PHP version 7.1.12, Laravel version 5.1, and Ionic version 3. The MTBB apps were developed on top of Ionic, which supports both iOS and Android. The MTBB server services were deployed on Amazon Web Services (AWS), which handles data security at the hardware, operating system, and Internet levels.

## MTBB design and implementation

### MTBB user roles and modules

The MTBB user interfaces have the user roles and modules shown in Table 3.

**Table 3:** Modules and roles in the MTBB

Entity	Role	Module
Member	Requestor, Provider	Vapp (Volunteer-use application on smartphones)
Organization	Coordinator	Oweb backend (Organization-use Web backend modules)
	Activity organizer	Oweb backend, Oapp (Organization-use application on smartphones)
	Financial specialist	Oweb backend
Superuser	Superuser managing all organizations and members	Tweb (Web backend modules for time-banking administration)

#### Member (Requestor, Provider) with Vapp

For members, Vapp is a mobile app that they can use to request elderly services for themselves or for elderly family member using time tokens; to provide elderly care services to earn time tokens; to sign in/out when delivering services; to inquire about service records and time tokens that are banked; to participate in volunteer activities held by organizations to earn time tokens; to enroll in organizations; to receive verification of service skills; to be eligible to provide services; and to receive app notifications in the app use scenarios, among other functions.

The push notification mechanism in mobile apps is the major reason for using a mobile app instead of a web page app. The appeal of the push notification mechanism is that app users can easily obtain real-time notifications for different mobile use scenarios.

#### Organization Coordinator with Oweb backend

The coordinator role, as illustrated by Cahn for 1–1 mutual service exchanges, has Oweb backend functions to monitor and assist members with their mutual service exchanges, including verifying members’ identity and skills, monitoring open service requests until requests are delivered, mediating the two sides of service requests and sending push app notifications.

#### Organization Activity Organizer with Oweb backend and Oapp

The Activity Organizer uses the Oweb backend to organize volunteer activities, enroll members, prepare sign-in/out services on volunteer activity days, calculate service time hours, and submit time token requests to the Financial Specialist in the organization, among other functions. The Activity Organizer can also use the Oapp mobile app to handle members’ sign-in/out actions on volunteer activity days.

#### Organization Financial Specialist with Oweb backend

The Financial Specialist uses the Oweb backend to review the time token requests from the Activity Organizer, approve and issue the time tokens. There is a separate role for handling time tokens for internal control reasons.

### MTBB system architecture

**Fig. 4:**
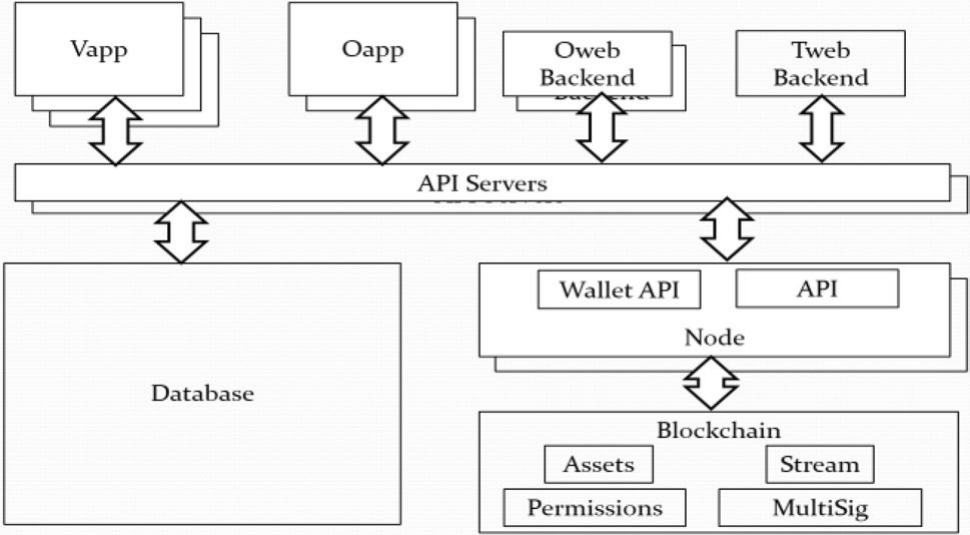
Technical architecture of the MTBB

**Fig. 5:**
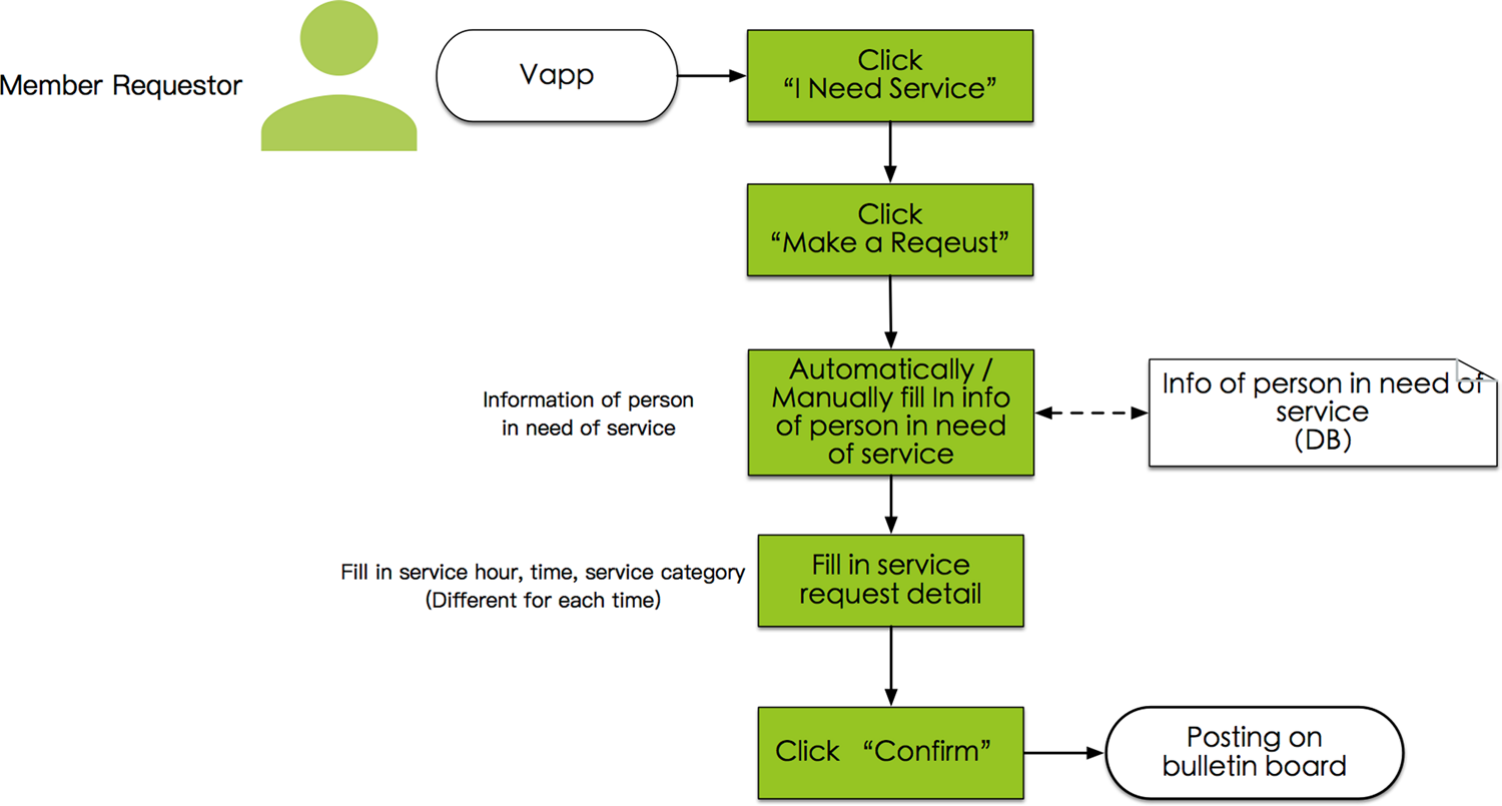
Service order request by the member requestor

**Fig. 6:**
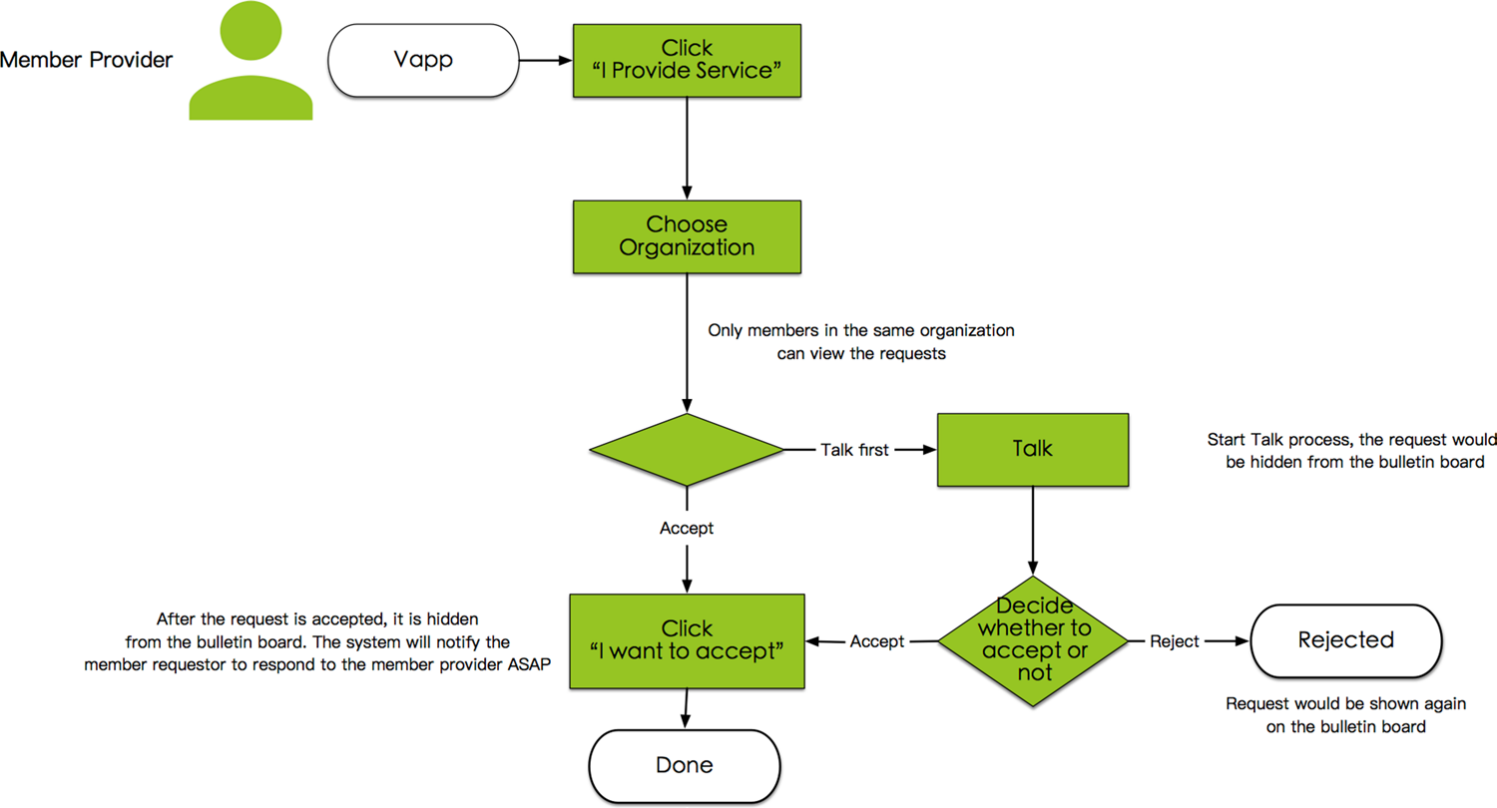
Service order response by a member provider

**Fig. 7:**
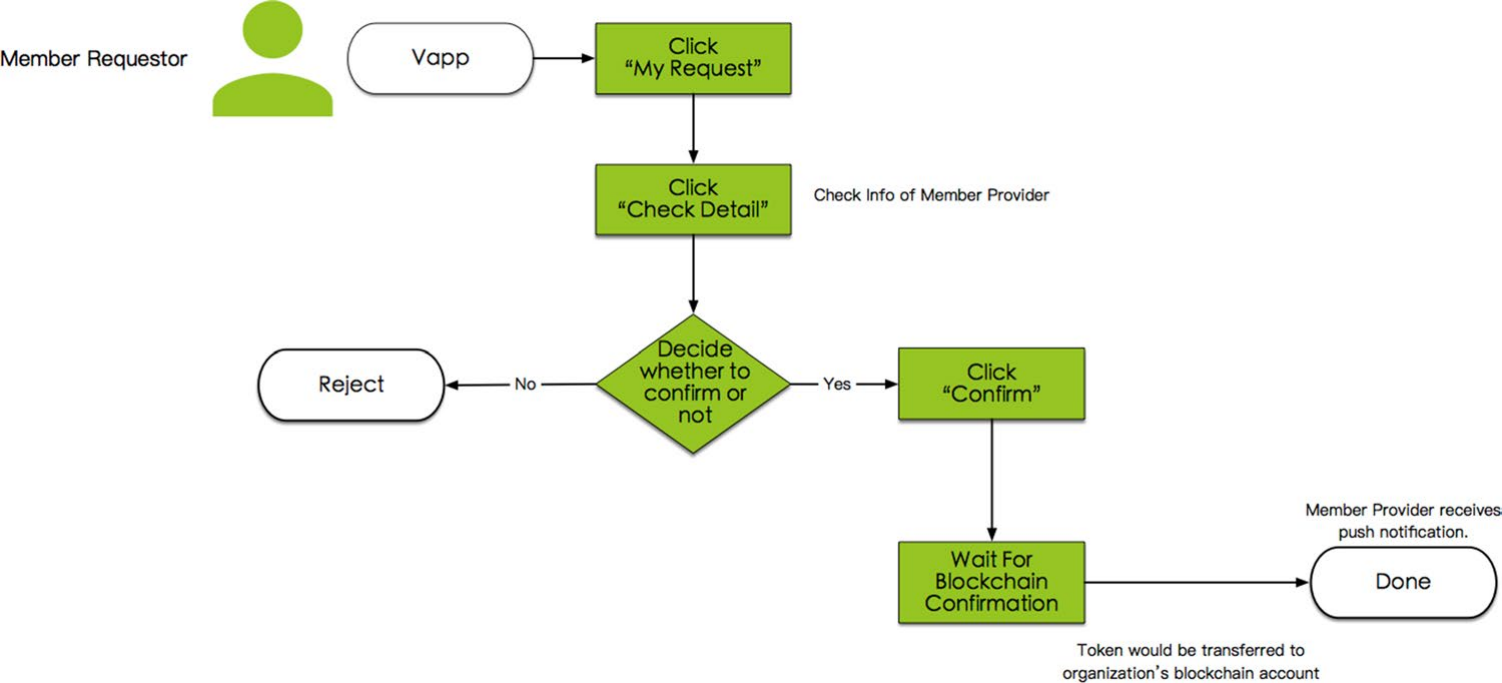
Service order confirmation by the member requestor

The high-level technical architecture diagram is shown in Fig. 4. The user interface modules are illustrated as previously described. All these modules access data via a layer of application programming interfaces (APIs) provided by API server instances. The APIs access both the database and the blockchain. The API calls are RESTful, and all of the communications over the Internet are conducted using the HTTPS protocol. The database is designed with Entity-Relationship diagrams to store all data, except time tokens. The included data contain log-in identities, members, skills, organizations, service categories, associations between organizations and members, service requests and statuses, and a variety of business process data, as illustrated in Figs. 5, 6, and 7. The blockchain stores time tokens in accordance with only the service transaction data.

### MTBB system process categories

From the business process perspective, the MTBB was designed to support the following five categories of business processes, as illustrated in Table 4.

**Table 4:** MTBB process categories

Process category	High level	Detail level
P1	Organization registration	Organization enrollment, Organization data management, Organization account management
P2	Organization member registration and verification	Member registration, Enrollment into an organization and Identity verification, Member skill verification, Member management
P3	Volunteer activity management by the organization	Volunteer activity creation, Member enrollment, Service day management, Time token plan and issuance
P4	Member mutual service exchange	Service request, Service order response, Service order confirmation, Service day management, Service backend management
P5	Ratings	Organization rating members, Member rating organizations, Member rating members

### MTBB process alignment with Cahn’s time-banking guidelines

With further expansion of the five categories of the system processes mentioned in Table 4, the MTBB was designed to follow Cahn’s twelve time-banking mechanism guidelines, as illustrated in Tables 5 and 6. For example, process P4.1, for member requests for service, aligns with guidelines TB2, TB5, and TB6 in Table 5 and guideline TB7 in Table 6.

**Table 5:** MTBB process alignment with the first six guidelines of Cahn’s time-banking guidelines

Time-banking guidelines MTBB business process	TB1 Members list the services they can offer and those that they need	TB2 All agree to both give and receive services	TB3 Everyone is interviewed and provides references	TB4 Every hour of giving help earns the giver one credit: a time dollar	TB5 Members 'buy' the services they need with their credits	TB6 The computer matches the task, the giver and the receiver
P1. New organization	X		X			
P2.1 New member						
P2.2 Member verification			X			
P2.3 Member skill verification	X	X	X			
P4.1 Member request for service		X			X	X
P4.2 Member response to request		X		X		X
P4.3 Member request confirmation		X			X	X
P4.4 Service check in		X		X	X	
P4.5 Service check out		X		X	X	
P4.6 Time token transfer				X	X	
P4.7 Org coordination						X
P4.8 Time token giveaway						
P3 Activity management	X		X	X		X
P5 Ratings

**Table 6:** MTBB process alignment with the final six guidelines of Cahn’s time-banking guidelines

Time-banking guidelines MTBB business process	TB7 Every transaction is recorded in a computer 'time bank'	TB8 Members receive a regular 'bank' statement	TB9 One hour is one credit regardless of the skills one offers	TB10 Members can donate credits to friends or to the 'credit pool'	TB11 Everyone is seen as special, with a contribution to make	TB12 All activities maintain set standards
P1. New organization						X
P2.1 New member						
P2.2 Member verification					X	
P2.3 Member skill verification					X	X
P4.1 Member request for service	X					
P4.2 Member response to request	X					
P4.3 Member request confirmation	X					
P4.4 Service check in	X					
P4.5 Service check out	X					
P4.6 Time token transfer	X	X	X			
P4.7 Org coordination	X					
P4.8 Time token giveaway	X	X		X		
P3 Activity management	X	X	X		X	X
P5 Ratings					X	X

### Example key business processes in the MTBB

Many business processes were designed in this MTBB. However, this paper illustrates only three key processes for the one-to-one member mutual service exchange scenario shown in Fig. 1 because they are the processes that will be used most often in local time-banking operations.

#### System process P4.1, service order request by the member requestor

In Vapp, the member service requestor selects the token type (associated with an organization) from among the token types that he/she previously earned; provides details of the request, including the date, time, hours, service category, and caretaker information; and then posts the request, as illustrated in Fig. 5.

#### System process P4.2, service order response by a member provider

After a service order request is posted on the private bulletin board that is associated with the organization, the MTBB sends out a push notification to all members within the same organization (with the token type). Only members in the same organization can see the requests on this private bulletin board in Vapp, which shows the list of all service requests posted by all member requestors in the same organization. One of the member providers sees the notification, opens Vapp, looks at the information on the service order request, uses the “talk” function to communicate further with the member requestor and then decides whether to accept the order by responding to it. The “talk” function is the texting tool in Vapp used to enable communication between the member requestor and the potential member provider. All communication texts are stored for managing potential customer service needs. If the member provider decides not to accept the order, the request will be reposted by the MTBB, as illustrated in Fig. 6.

#### System process P4.3, service order confirmation by the member requestor

After the member provider responds, the MTBB sends a push notification to the requestor. The requestor then opens the Vapp, looks at the information about the member provider, and confirms the response from this member provider. If the requestor is not willing to receive a service from the member provider, the MTBB will repost the service order request for the member requestor, as illustrated in Fig. 7.

After this service-ordering process is completed, the associated time tokens are transferred to the MTBB and placed in escrow. On the service date, after the service is completed, the time tokens are transferred to the member provider from the MTBB. If the service is not performed or is performed at an unacceptable level, the time tokens are refunded and transferred to the member requestor via the customer service process. After the service is completed, neither the member requestor nor provider can see the details of each other’s member information for security considerations.

### Integration with blockchain technology

In the blockchain integration, the service transaction and token data are categorized into three types of encryption security levels; for the first type, the token type and token expiration date are unencrypted. The second type of encryption, which can be privately viewed, includes the database schema primary keys which allow the system superuser to access the database for auditing purposes. The last type of encryption allows the attributes to be publicly viewed, this type allows outside processing so that organizations can analyze service and time token transactions. The attributes are shown in Table 7. For security considerations, member personal data, except the de-tagged name, are not stored on blockchain.

**Table 7:** Service record attributes in the MultiChain blockchain

Attribute name	Meaning
FromWhom	Time token transfer from whom, de-tagged
ToWhom	Time token transfer to whom, de-tagged
Date	Service date for the time token transfer
Time	Service time for the time token transfer
Hours	Hours of service time length for the time token transfer
TokenType	Proprietary token type for the time token transfer
Event	Time token transfer at the event, such as initial issuance, service, gift, expiration, conversion, etc.
ServiceCategory	Elderly care service category, home or community
ServiceType	Service type, i.e., CC1–CC11, HC1–HC9
ServiceSubtype	One-level-down detail under ServiceType
ServiceArea	Area of the elderly care service, 3 levels (state, county, and city)
CaregiverGender	Gender of the service caregiver for the time token transfer
CaregiverAge	Age of the service caregiver for the time token transfer
CaregiverResidenceArea	Residence area of the service caregiver, 3 levels (state, county, and city)
ifGroup	Whether the time token transfer occurs from a volunteer activity
GroupSize	If the transfer occurs from a volunteer activity, the volunteer group size (number of people involved)
ifIndividual	Whether the time token transfer occurs for a one-to-one mutual service exchange
ReceiverGender	Gender of the service receiver for the time token transfer
ReceiverAge	Age of the service receiver for the time token transfer
ReceiverResidenceArea	Residence area of the service receiver, 3 levels (state, county, and city)

This MTBB design also introduces the expiration of time tokens to promote time token circulation. Without token expiration, members may keep time tokens for a long time. To avoid time token expiration, members can give their tokens to other members of the same organization.

The organizations which use the MTBB are responsible for validating the service records from the business perspective and conducting transactions. There are Oweb backend modules for the organizations to monitor and manage the transactions within the organizations from the business perspective. There are also Tweb backend modules for the system business operator (Superuser role described in Table 3) to manage all the transactions of the organizations and members across all organizations. The MTBB is hosted as SaaS on AWS by the GCEC Taiwan company and the blockchain is handled as a private blockchain.

The blockchain smart contract mechanism is not used in the design because in the real world, many customer service scenarios are not preplanned when the smart contract is initially designed and deployed. After the smart contract is deployed, there is no way to change it, which presents challenges in system deployment. Furthermore, some customer services may be handled as exceptions to satisfy both sides, and those appeasements are not entirely rule-based decisions.

### MTBB elderly care service categories

In addition to the system design, there are two types of service categories: communities and homes. The difference is where the service occurs; usually, the community type refers to services that are provided in senior living communities, and the home type refers to services that are provided in individuals’ homes.

#### Community elderly care service categories

There are eleven service categories for community elderly care (CC). These service categories cover care for elderly people or their family members in communities and community administrative tasks, as shown in Table 8. In Taiwan, these service categories are common practices which originated in the Taiwan long-term elderly care welfare system (Department of Health 2019).

**Table 8:** Community elderly care service categories

Community care category number	Description
CC 1	Healthy diet
CC 2	Health improvement
CC 3	Community care companionship
CC 4	Transportation services
CC 5	Physical care
CC 6	Nursing services
CC 7	Mental comfort
CC 8	Support to family caregivers
CC 9	Community affairs
CC 10	Care phone calls to the elderly
CC 11	Resource connections in the community

#### Home elderly care service categories

There are nine service categories in elderly home care (HC). These service categories cover care for elderly people or their family members in homes and care-related housekeeping tasks, as listed in Table 9. In Taiwan, these service categories are common practices which originated in the Taiwan long-term elderly care welfare system (Department of Health 2019).

**Table 9:** Home elderly care service categories

Home care category number	Description
HC 1	Healthy diet
HC 2	Health improvement
HC 3	Home companionship
HC 4	Transportation services
HC 5	Physical care
HC 6	Home nursing
HC 7	Mental comfort
HC 8	Support for family caregivers
HC 9	Housekeeping

### MTBB elderly care service category alignment with the SDGs of the UN

The elderly care service categories were designed to align with the UN’s SDGs. The alignment for community elderly care is illustrated in Table 10, and the alignment for home elderly care is illustrated in Table 11. For example, CC1 healthy diet in Table 10 aligns with SDG #1 (if the member that receives services is in poverty), SDG #2 (self-explanatory), SDG #3 (because elderly care aims to promote healthy living for the elderly), SDG #4 (because members learn how to provide healthy diets to members), and SDG #5 (if the member that provides services is male). The other forms of alignment are self-explanatory.

**Table 10:** Alignment between elderly community care service categories and SDGs

SDGs Community care service categories	SDG #1, End poverty	SDG #2, End hunger	SDG #3, Ensure healthy lives	SDG #4, Promote lifelong learning opportunities	SDG #5, Achieve gender equality and empower all women and girls
CC1. Healthy diet	X	X	X	X	X
CC2. Health improvement			X	X	X
CC3. Community care companionship			X	X	X
CC4. Transportation services	X		X	X	X
CC5. Physical care			X	X	X
CC6. Nursing service			X	X	X
CC7. Mental comfort			X	X	X
CC8. Support to family caregivers			X	X	X
CC9. Community affairs				X	X
CC10. Care phone calls to the elderly			X	X	X
CC11. Resource connections in the community				X	X
	* if the member that receives services is in poverty				* if the member that provides services is male

**Table 11:** Alignment between elderly home care service categories and SDGs

SDGs Home care service categories	SDG #1, End poverty	SDG #2, End hunger	SDG #3, Ensure healthy lives	SDG #4, Promote lifelong learning opportunities	SDG #5, Achieve gender equality and empower all women and girls
HC1. Healthy diet	X	X	X	X	X
HC2. Health improvement			X	X	X
HC3. Home companionship			X	X	X
HC4. Transportation services	X		X	X	X
HC5. Physical care			X	X	X
HC6. Home nursing			X	X	X
HC7. Mental comfort			X	X	X
HC8. Support to family caregivers			X	X	X
HC9. Housekeeping	X			X	X
	* if the member that receives services is in poverty				* if the member that provides services is male

## Rollouts and discussion

In this study, based on modern blockchain technology and Cahn’s time-banking guidelines, the MTBB for tracking elderly care service transaction records was developed, hosted as SaaS by the GCEC Taiwan company and rolled out to the selected organizations in Taiwan, as shown in Table 12.

**Table 12:** MTBB rollouts in Taiwan by December of 2020

Organization short name	Number of volunteer activities held	Number of volunteer attendances	Number of time tokens issued
L	1	6	27
A	3	44	120
C	8	137	323

The first rollout is Organization L, which provides community elderly care services in Northern Taiwan. Organization L recruits social workers and community volunteers. In June 2019, this organization employed the MTBB to manage one community care companionship activity and issued 27 proprietary time tokens with 6 attendances for trial. Afterwards, Organization L determined that it would have to reconsider the rollout strategy because most of its volunteers are more than seventy years old and their concurrent use of the system would be difficult. The second organization is Organization A, which is a nursing-home-like institute in Northern Taiwan. Organization A recruits community volunteers to accompany senior people who live in the institute. Between June and August 2019, this organization employed the MTBB to manage three activities and issued 120 proprietary time tokens with 44 attendances. Due to the COVID-19 pandemic, all nursing-home-like institutes are under strict control and volunteer activities have been suspended. Another organization is Organization C, which is a science-and-technology college in Central Taiwan that provides elderly caregiving education and training. Organization C recruits student volunteers and community volunteers in rolling out health improvement activities for the senior people who live in the communities around the college campus. Between May and December 2020, Organization C utilized the MTBB to manage eight activities and issued 323 proprietary time tokens with 137 attendances. There were some one-to-one member mutual service exchanges. Organization C plans to promote additional one-to-one member mutual service exchanges after additional community activities are held in subsequent months.

In this study, decentralization is not the first-priority blockchain feature employed because the decision to host the applications on AWS precludes any decentralization, that is, it is no longer a decentralized approach. In addition, considering that not all the organizations in the real world may be capable of running the blockchain nodes for the so-called decentralization, the decision was made to initially forgo the decentralization blockchain feature. However, multiple nodes could be deployed by different organizations in the future. In contrast, the immutability feature is mainly employed as the first step because the records on blockchain cannot be altered after the records are generated. While there are always debates between using blockchain technology and using database technology, this study has attempted to explore a new way to apply the blockchain technology to elderly care in the real world.

In this study, blockchain scalability is addressed from the business process perspective, but not from the technology perspective. The business process of issuing time tokens requires the approval of the organization’s financial specialist and afterwards, the system performs batch jobs asynchronously to handle the transactions on blockchain. In the one-to-one member mutual service exchange scenario, as described in Sect. 4.5, since the number of transactions is not huge due to the limited rollout, the system performance is acceptable. In the near future, adding the capability of the underlying technology stack in the hosted environment on AWS to prevent performance bottleneck is planned.

## Conclusions

In this study, the MTBB was designed, implemented, and rolled out. Blockchain technology was integrated in the system to track elderly care service records, which enables corporations to align their corporate-social-responsibility activities with the SDGs of the UN.

When the paper was submitted, this MTBB was rolled out to only a few organizations mostly due to the COVID-19 global pandemic. However, given its framework and the detailed business processes designed for construction and validation of the MTBB by following Cahn’s twelve time-banking guidelines, the system can be easily rolled out on a broader scale in the future. Therefore, incorporating these rollout data and experiences will make our next-generation MTBB more compelling and robust.
